# Primary and secondary non-adherence to osteoporotic medications after hip fracture in Spain. The PREV2FO population-based retrospective cohort study

**DOI:** 10.1038/s41598-017-10899-6

**Published:** 2017-09-18

**Authors:** Aníbal García-Sempere, Isabel Hurtado, José Sanfélix-Genovés, Clara L. Rodríguez-Bernal, Rafael Gil Orozco, Salvador Peiró, Gabriel Sanfélix-Gimeno

**Affiliations:** 1 Centro Superior de Investigación en Salud Pública (CSISP-FISABIO), Valencia, Spain; 2 Red de Investigación en Servicios de Salud en Enfermedades Crónicas (REDISSEC), Valencia, Spain; 3 Centro de Salud de Nazaret, Instituto de Investigación Sanitaria INCLIVA, Valencia, Spain; 4 Servicio de Medicina Preventiva, Hospital de Vinaroz, Castellon, Spain

## Abstract

Osteoporotic medication after hip fracture is widely recommended by clinical practice guidelines, and medication adherence is essential to meet clinical trial risk reduction figures in the real world. We assessed primary and secondary non-adherence to osteoporosis medications in patients discharged following a hip fracture and identified factors associated with secondary non-adherence. From a population-based retrospective cohort of 19,405 patients aged 65 years and over discharged from a hip fracture in the region of Valencia (Spain) from January 1, 2008 and June 30, 2012, we followed, over a minimum of 365 days, 4,856 patients with at least one osteoporotic medication prescribed within the first six months after discharge. Less than one third of the patients discharged alive after a hip fracture received osteoporotic treatment. Primary non-adherence among naïve patients was low. However, long-term secondary adherence measured by Proportion of Days Covered with medication (PDC) and persistence was largely suboptimal, with naïve users having worse results than experienced patients. Secondary non-adherence was associated with primary non-adherence and age, dementia or sedative treatments for naïve users and with being male, being older than 85 and having dementia for experienced users. Three quarters of naïve users and two thirds of experienced users had interrupted treatment at 48 months.

## Introduction

Osteoporotic fractures (also known as fragility or low-trauma fractures) commonly occur in three main sites –vertebrae column, wrist and hip– and result in significant reductions in quality of life, disability, morbidity^1,2^ and mortality^3^, which translate into considerable costs to health care systems^4^. Hip fractures are the worst consequence of osteoporosis, as patients’ one-year mortality after a hip fracture is nearly 30%, accompanied by major morbidity, significant functional loss and worsening of quality-of-life^5^. In Spain, the annual incidence of hip fracture is estimated at 72 and 28 per 10,000 women and men respectively, totalling roughly 40,000 hip fractures/year^6^. Moreover, the age and sex standardised rate of hip fracture per 10,000 inhabitants has remained relatively stable in the period 2000–2012^7^ despite significant increments in osteoporotic medication prescription in the Spanish National Health System.

While pharmacological primary prevention in people with a low risk of fracture is highly controversial^8^, secondary prevention treatment after hip fracture with bisphosphonates or other alternative drugs is recommended by almost every clinical practice guideline^9^. Nevertheless, several studies show post-fracture treatment remains suboptimal^10^, and only 20% to 30% of patients receive one anti-osteoporosis drug after a hip fracture. Underuse does not seem to improve over time^11^, and despite showing large variations^12^, it affects geographical areas with very different health care systems^13^.

Additionally, even when osteoporosis treatment is prescribed, the issue of non-adherence arises. Medication adherence is essential to achieve clinical trial risk reduction figures in the real world, and although there is strong evidence on some of the factors associated with discontinuation or low adherence (risk perception, perceived benefits and disadvantages of drugs, self-efficacy, communication problems with physicians, etc.)^14^, our ability to characterize non-adherent patients is limited. Gaining knowledge of the factors associated with medication non-adherence to osteoporotic treatments may allow healthcare organisations to identify non-adherent patients early on and to design personalized interventions to improve their management. Furthermore, the study of primary non-adherence (not filling the first prescription), an essential but under-analysed phenomenon, may help to better understand the whole process of medication adherence.

The aim of this study is to assess primary and secondary non-adherence to osteoporosis medications in patients discharged after a hip fracture and to identify factors associated with secondary non-adherence.

## Methods

### Design

Population-based retrospective cohort of all patients aged 65 years and over discharged following hip fracture hospitalisation from January 1, 2008 to June 30, 2012, who were prescribed at least one osteoporotic medication within the first six months after discharge and who were followed for a minimum of 365 days and a maximum of 5.5 years from the date of discharge (end of the study June 30, 2013).

### Setting

The study was conducted in the region of Valencia in Spain (5.1 million inhabitants registered population in 2010) and, specifically, in the Valencia Health System (VHS), an extensive network of public hospitals, primary care centres and other facilities managed by the regional government, which provides universal free healthcare services (except for drug copayment) to 97% of the regional population.

### Population

We identified all patients aged 65 and over discharged alive from VHS hospitals with a main diagnosis of hip fracture (International Classification of Diseases 9^th^ revision Clinical Modification [ICD9CM] codes: 820.xx and 733.14) and no diagnosis of multiple fracture or road accident between January 1, 2008 and June 30, 2012, who had received at least one osteoporotic treatment within 6 months after discharge. Patients were classified for analysis as naïve or experienced users based on the presence or not of osteoporotic medication within a year before the hip fracture, as evidence suggests that those groups behave differently with regards to adherence. We followed them for a minimum of 365 from the date of discharge (index date). Patients were censored at disenrollment from the VHS insurance, hospitalisation for a new hip fracture, death or the end of the study. Exclusion criteria were dying within the first month after the index date or being non-resident in the region of Valencia.

### Data sources

Information was obtained from the electronic information systems of the VHS. The Population Information System (SIP) provides information on the population under VHS coverage and registers some demographic characteristics, including the geographical/contextual situation of every person and dates and causes of VHS discharge, including death. The Minimum Basic Dataset (MBDS) at hospital discharge is a synopsis of clinical and administrative information on all hospital discharges, including diagnoses and procedures. The electronic medical record for ambulatory care (EMR), available in all primary healthcare centres and ambulatory facilities, has information about diagnoses, personal and family medical history, laboratory results, lifestyle, etc. and information about both physician prescriptions and dispensations from pharmacy claims. All the information in these systems is linked at an individual level through a unique identifier.

### Study endpoints

We assessed primary and secondary non- adherence (proportion of days covered [PDC] and persistence) to osteoporosis medications one year and four years after the first prescription following discharge. Primary non-adherence was defined as not filling the index prescription after discharge (a naïve patient not filling the first prescription would be considered as primary non-adherent)^15^. PDC was calculated by dividing the number of days of medication dispensed by the number of days of the follow-up period, and was summarized categorically as adherent/fully adherent (PDC ≥ 80%), partially adherent (20% < PDC < 80%), or non-adherent (PDC < 20%). Non-persistence was defined as the interruption of the use of osteoporosis medications from the index prescription when exceeding a 90-day permissible gap^16,17^. Osteoporosis drugs considered included oral bisphosphonates (alendronate, risedronate, ibandronate and etidronate), raloxifene, strontium ranelate, calcitonin nasal spray, teriparatide, parathyroid hormone and denosumab. Zoledronic acid was not included because in Spain it is administered in-hospital, and medications dispensed in-hospital are not captured by the EMR.

### Covariates

Variables potentially related to the risk of hip fracture and to the use of osteoporosis medication were assessed using data from a 365-day baseline period before the index hospitalisation. These included socio-demographic characteristics, severity, comorbidities, pre-fracture use of osteoporosis medication and other concomitant drugs, and health care utilization factors.

### Ethics approval and consent to participate

The study, observational in design and with retrospective data anonymized prior to their transfer to the research team, was approved by the Ethics Committee for Clinical Research of the General Directorate of Public Health and the Centre for Public Health Research (session on October 26, 2012). All methods were performed in accordance with the relevant guidelines and regulations. Patient informed consent exemption was approved by the aforementioned Committee given the nature of the study. All patient data were transferred to the research team anonymized and de-identified prior to analysis according to Spanish laws on privacy (Act 15/1999) and patient’s rights (Act 41/2002).

### Analysis

We first described baseline patient characteristics. We calculated primary non-adherence, PDC and persistence to medication from the index date. Kaplan-Meier curves were constructed to estimate the distribution of the time until a patient exceeded a permissible gap of 90 days without medication available. Differences between the survival curves were assessed by using a long-rank test. We conducted multivariable regression models to determine which covariates were associated with secondary non-adherence, both in new and experienced users, by means of multivariable logistic regression models (for non-adherence, PDC < 20%) and Cox regression models (for non-persistence). We included all covariates in the multivariable models and used backward-stepwise methods (with a removing probability of 0.10 and an entry probability of 0.05) to retain the significant variables. The corresponding odds ratios (OR) and hazard ratios (HR) with their 95% confidence intervals were estimated. Predictive capacity was assessed by means of the C-statistic. All the analyses were performed using the Stata 13.0 (Stata Corp) statistical software.

## Results

### Patient characteristics at baseline

**Figure 1:**
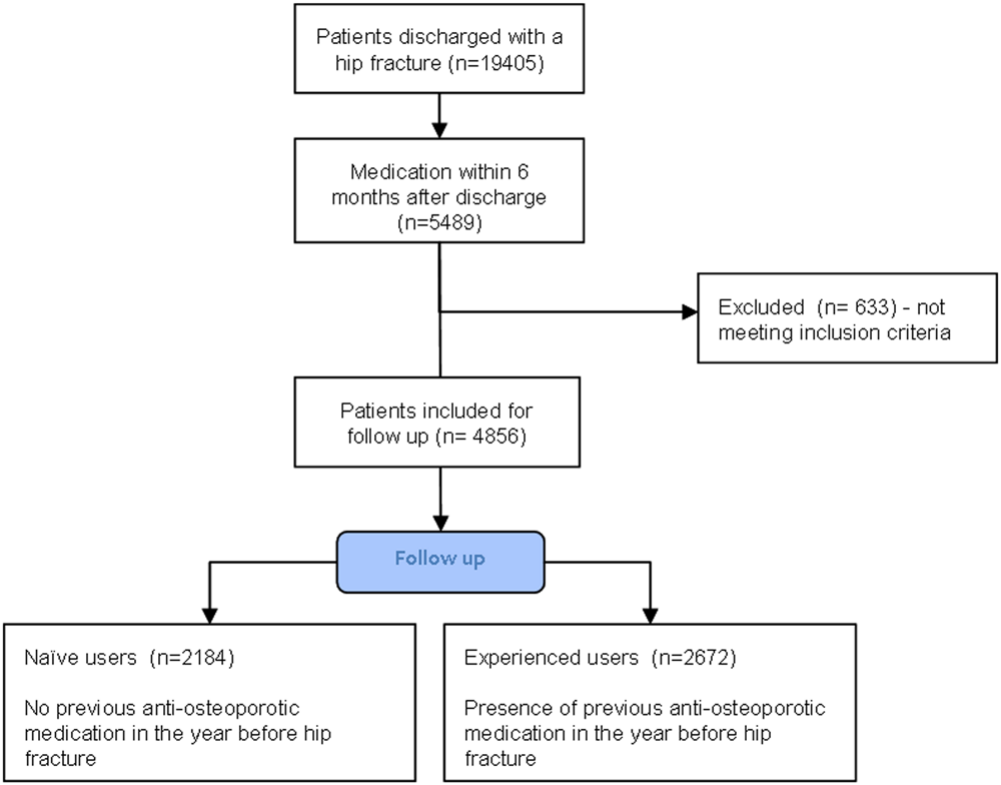
Study flowchart.

From the 19,405 patients discharged alive after a hip fracture, 5,489 (28.3%) were prescribed an osteoporotic medication within a period of 6 months (Fig. 1). After taking into account the exclusion criteria, a cohort of 4,856 patients classified as naïve (n = 2,184, 45%) or experienced users (n = 2,672, 55%) was established (average follow-up was 35 months for naïve users and 32 months for experienced users).

Patients were mostly women (87.4%) and a majority used more than 6 different medications (76.7% and 90.9% for naïve and experienced users, respectively). Naïve users (Table 1) were older than experienced users (34.6% aged 85 and over vs. 29.5%), the proportion of men was almost double (16.9% vs. 9.2%), and they were less likely to have previous fractures (13.1% vs. 23.5%), to use the healthcare services (except for hospital visits for which non-significant differences were found) and to use concomitant medication (19.2% taking more than 13 drugs vs. 33.4%). Comorbidities were similar among groups except for diabetes and stroke, which were more prevalent in new users (30.0% and 9.9% in naïve vs. 24.7% and 8.1% in experienced users, respectively) and for osteoporosis (5.5% vs. 27.9%) and rheumatoid arthritis (2.3% vs. 3.6%), which were more prevalent in experienced users. Experienced patients were prescribed more opioids (19.3% vs. 34.4%), sedative agents (51.8% vs. 58.1%) and serotonin reuptake selective inhibitors (19.3% vs. 26.4%).

**Table 1:** Baseline characteristics of naïve and experienced users of osteoporotic medication after hip fracture.

Characteristics		Naïve (n = 2,184)		Experienced (n = 2,672)		Total (n = 4,856)	%	p value
		N	%	N	%	N		
**Socio-demographic**								
Gender	women	1,815	83.1	2,427	90.8	4,242	87.4	<0,001
	men	369	16.9	245	9.2	614	12.6	
Age	65–74	312	14.3	422	15.8	734	15.1	0.001
	75–84	1,117	51.1	1,464	54.8	2,581	53.2	
	≥ 85	755	34.6	786	29.4	1,541	31.7	
**Severity**								
Previous fracture	Yes	285	13.1	627	23.5	912	18.8	<0,001
	No	1,899	86.9	2,045	76.5	3,944	81.2	
Charlson Index	0	899	41.2	1,149	43.0	2,048	42.2	0.357
	1	631	28.9	766	28.7	1,397	28.8	
	>= 2	654	29.9	757	28.3	1,411	29.1	
**Health Services Use**								
Primary care visits	0–4	537	24.6	372	13.9	909	18.7	<0,001
	5–12	810	37.1	910	34.1	1,720	35.4	
	13	837	38.3	1,390	52.0	2,227	45.9	
ER visits	0	1,367	64	1,598	59.8	2,995	61.7	0.008
	1	428	19.6	559	20.9	987	20.3	
	>= 2	359	16.4	515	19.3	874	18.0	
Hospital visits	Yes	382	17.5	521	19.5	903	18.6	0.074
	No	1,802	82.5	2,151	80.5	3,953	81.4	
Polypharmacy	0–5	508	23.3	243	9.1	751	15.5	<0,001
	6–12	1,256	57.5	1,539	57.6	2,795	57.6	
	≥13	420	19.2	890	33.3	1,310	27.0	
**Comorbidities**								
Osteoporosis	Yes	121	5.5	746	27.9	867	17.9	<0,001
	No	2,063	94.5	1,926	72.1	3,989	82.2	
Parkinson	Yes	94	4.3	136	5.1	230	4.7	0.200
	No	2,090	95.7	2,536	94.9	4,626	95.3	
Dementia	Yes	348	15.9	425	15.9	773	15.9	0.979
	No	1,836	84.1	2,247	84.1	4,083	84.1	
Diabetes	Yes	655	30	658	24.6	1,313	27.0	<0,001
	No	1,529	70	2,014	75.4	3,543	73.0	
Rheumatoid Artritis	Yes	51	2.3	95	3.6	146	3.0	0.013
	No	2,133	97.7	2,577	96.4	4,710	97.0	
Stroke	Yes	217	9.9	215	8.0	432	8.9	0.021
	No	1,967	90.1	2,457	92.0	4,424	91.1	
Myocardial infarction	Yes	199	9.1	236	8.8	435	9.0	0.735
	No	1,985	90.9	2,436	91.2	4,421	91.0	
Heart failure	Yes	164	7.5	183	6.9	347	7.2	0.374
	No	2,020	92.5	2,489	93.1	4,509	92.9	
Cancer	Yes	212	9.7	303	11.3	515	10.6	0.066
	No	1,972	90.3	2,369	88.7	4,341	89.4	
**Medication**								
Osteoporosis–injectable	Yes	286	13.1	148	5.5	434	8.9	<0,001
	No	1,898	86.9	2,524	94.5	4,422	91.1	
Opioid treatment	Yes	421	19.3	918	34.4	1,339	27.6	<0,001
	No	1,763	80.7	1,754	65.6	3,517	72.4	
Sedative treatment	Yes	1,131	51.8	1,551	58.1	2,682	55.2	<0,001
	No	1,053	48.2	1,121	41.9	2,174	44.8	
SSRI treatment	Yes	421	19.3	705	26.4	1,126	23.2	<0,001
	No	1,763	80.7	1,967	73.6	3,730	76.8	

ER: Emergency room; SSRI: Selective Serotonin Reuptake Inhibitors.

### Primary and secondary non-adherence at 1 and 4 years of follow-up

**Figure 2:**
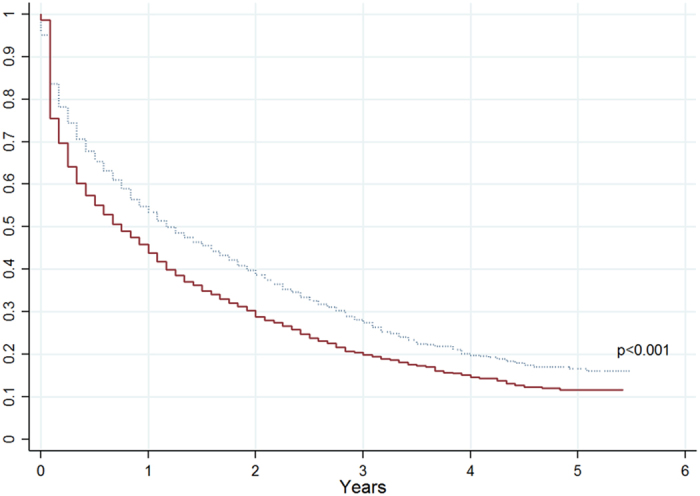
Persistence with treatment stratified by naïve and experienced users. Solid line: naïve users; Dotted line: experienced users.

Primary non-adherence expressed as a percentage of naïve patients that did not fill their first prescription after discharge was 2.8%. Mean PDC at 12 months was 58.7% for naïve and 66.6% for experienced users. At 4 years, mean PDC declined to 46.3% and 57.3%, respectively. The percentage of non-adherent patients (PDC < 20%) was 25.2% and 14.9% for naïve and experienced users at 12 months respectively, rising to 33.9% and 20.8% at 48 months. One year after the event, 54.5% of naïve patients had discontinued osteoporosis treatment (non-persistence rate) vs. 44.9% of experienced users, rising to 75.4% and 67.1% respectively after 4 years (Table 2). Persistence was different for naïve and experienced users for the whole period (p < 0.001, Fig. 2). However, those differences arose at the first two years of the follow-up period (p < 0.001) and no differences in persistence were observed from the third year to the end of follow-up (p = 0.6882).

**Table 2:** Secondary adherence at 1 and 4 years of follow-up.

		1 year		4 years	
		Naïve users Mean/% (95CI)	Experienced users Mean/% (95CI)	Naïve users Mean/% (95CI)	Experienced users Mean/% (95CI)
PDC (mean)		58.7 (57.2–60.2)	66.6 (65.4–67.8)	46.3 (44.8–47.8)	57.3 (56.0–58.5)
PDC (%)	<20%	25.2 (23.5–27.1)	14.9 (13.6–16.3)	33.9 (31.9–35.9)	20.8 (19.2–22.4)
	20–80%	32.1 (30.2–34.1)	35.7 (33.9–37.5)	39.9 (37.8–42.0)	43.5 (41.6–45.3)
	≥80%	42.6 (40.6–44.7)	49.4 (47.5–51.3)	26.2 (24.4–28.1)	35.8 (34.0–37.6)
Non–persistence (%)		54.5 (52.4–56.6)	44.9 (43.0–46.8)	74.7 (72.9–76.5)	66.5 (64.7–68.2)

PDC: Proportion of Days Covered; 95CI: 95% confidence intervals for mean or proportions.

### Factors associated with secondary non-adherence at 1 year

In multivariable analysis (Table 3), primary non-adherence was associated with 2.64 greater odds of non-adherence and with 2.26 greater odds of non-persistence among naïve users. Ageing was also associated with non-adherence (OR: 1.51 and 2.14 for patients who were 75–84 years old and patients who were 85 years or older, respectively, compared to patients who were 65–74 years old), and non-persistence (HR: 1.25 and 1.61 for patients who were 75–84 years old and patients who were 85 years or older, compared to patients who were 65–74 years old) among naïve patients. In this group of patients, sedative treatment and previous stroke were also associated with poor adherence (OR: 1.31 and 1.38 respectively) while being male (HR: 1.25) and having dementia (HR: 1.18) impaired persistence. Polypharmacy was associated with better PDC (OR: 0.61 for 6 to 13 drugs and 0.65 for 13 drugs or more, compared to using 0 to 5 drugs) and persistence (HR: 0.79 for patients with 6 to 13 drugs but non-significant for patients with 13 drugs or more, compared to using 0 to 5 drugs).

**Table 3:** Factors associated with secondary adherence at 1 year. Multivariable logistic regression analysis for low adherence (PDC < 20%) and Cox regression for non-persistence (>90 days gap).

		Non adherence (PDC < 20%)		Non persistence (>90 days gap)	
		Naïve users	Experienced users	Naïve users	Experienced users
Sex	Women	—	1	1	1
	Men	—	1.89 (1.37–2.60)	1.25 (1.08–1.46)	1.42 (1.18–1.71)
Age	65 –74	1	1	1	1
	75–84	1.51 (1.09–2.09)	1.32(0.93–1.89)	1.25 (1.04–1.51)	1.10 (0.93–1.31)
	>= 85	2.14 (1.53–2.99)	2.17(1.50–3.13)	1.61(1.39–1.95)	1.43 (1.19–1.72)
Primary non–adherence	Adh.	1	na	1	na
	Non–adh.	2.64 (1.57–4.46)	na	2.26 (1.66–3.07)	na
Polypharmacy	0–5	1	1	1	—
	6–12	0.61 (0.48–0.79)	0.55 (0.39–0.77)	0.79 (0.69–0.91)	—
	>= 13	0.65 (0.47–0.89)	0.39 (0.27–0.58)	0.87 (0.74–1.05)	—
Charlson Index	0	—	1	—	—
	1	—	1.16 (0.88–1.51)	—	—
	>= 2	—	1.33 (1.01–1.73)	—	–
ER visits	0	—	1	—	—
	1	—	0.85 (0.64–1.14)	—	—
	>= 2	—	1.24 (0.93–1.65)	—	—
Sedative	No	1	—	—	—
treatment	Yes	1.31 (1.06–1.62)	—	—	—
Stroke	No	1	—	—	—
	Yes	1.38 (1.01–1.89)	—	—	—
Dementia	No	—	—	1	1
	Yes	—	—	1.18 (1.02–1.38)	1.31 (1.13–1.53)
Diabetes	No	—	1	—	
	Yes	—	1.27 (0.98–1.64)	—	
Osteoporosis diagnosis	No	—	1	—	1
	Yes	—	0.69 (0.53–0.90)	—	0.86 (0.75–0.98)
Rheumatoid arthritis	No	—	—	–	1
	Yes	—	—	—	0.67 (0.46–0.97)
C-statistic		0.60	0.65	0.58	0.57

Values are Odds Ratios for non-adherence (logistic regression models) and Hazard Ratios for non-persistence (Cox regression models) with the corresponding 95% confidence intervals between brackets. PDC: Proportion of Days Covered. All covariates in Table 1 were included in the multivariable models. Some covariates are not presented because of non-significance. Blank cells also represent non-significant associations. Analysis for experienced users is modelled excluding primary non-adherence.

For experienced users, being a male and age (but only being older than 85) were associated with both non-adherence (OR: 1.89 for being male and OR: 2.17 for being 85 years old or older) and non-persistence (HR: 1.25 for being male and OR: 1.43 for being 85 years old and older). Polypharmacy was associated with lower non- adherence (OR: 0.55 and 0.39 for 6 to 13 and more than 13 drugs, respectively), while dementia affected persistence negatively (HR: 1.31). The presence of osteoporosis was associated with lower non-adherence and non-persistence in experienced users (OR: 0.69; HR: 0.86, respectively) and the same happened for a diagnosis of rheumatoid arthritis regarding non-persistence (HR: 0.67, see Table 3). Predictive capacity was low, with the C-statistic ranging from 0.57 to 0.65.

## Discussion

To the best of our knowledge, this is one of the very few studies providing evidence on the complete view of adherence to osteoporosis treatment after hip fracture for patients with no previous osteoporotic medication and for prevalent users. We found that less than one third of patients discharged alive after hip fracture received osteoporotic treatment within six months of discharge. Also, even if primary non-adherence among naïve patients was low (when compared to studies in patients with a new prescription of oral biphosphonates, not necessarily newly fractured)^18^, secondary non - adherence measured by PDC and persistence was prevalent in both groups with a higher percentage in new users than in experienced patients. Non-adherence and non-persistence increased over time in both groups, with half of the patients having interrupted medication at 12 months, this proportion rising to three quarters of naïve users and two thirds of experienced users at 48 months.

Experienced users were younger but had more comorbidity, used more previous medication and health services and had more previous fractures, configuring a relatively riskier group than new users. Regarding factors associated with non-adherence, new-user figures worsened when some risk factors increased (age, dementia and sedative treatments). For experienced users, those with previous osteoporosis or rheumatoid arthritis were more likely to be adherent, while being male, being older than 85 and having dementia were associated with lower PDC and persistence. Polypharmacy was associated with better adherence and persistence in both groups. This is an unusual finding as usually an increasing number of medications are linked to a decreased adherence.

The low rate of patients with medication prescribed after hip fracture is a consistent finding in the literature and raises concerns about adherence to clinical practice guidelines by our healthcare providers and possible strategies for improvement^19,20^. In the case of osteoporotic fractures, reasons for this insufficient adherence to guidelines should be investigated and appropriate policies should be put in place.

We found a low rate of primary non-adherence that may be explained by raised patient awareness due to the proximity of a very severe event such as a hip fracture. However, many naïve patients discontinued treatment with just over 50% maintaining it at 6 months. Thus, even if primary non-adherence was strongly associated with secondary non-adherence in naïve patients, other factors are further impeding the achievement of desirable long-term adherence rates. Older patients with dementia, especially men, appear to be particularly vulnerable, but other factors not considered in our study (adverse effects, profile of caregivers or deficient information about risks) may well be equally important. For example, experienced users with a previous diagnosis of osteoporosis or rheumatoid arthritis – and thus with acknowledged risks - were less likely to be non-adherent. Beliefs and attitudes of health professionals towards osteoporotic treatments can also influence patient adherence behaviour. Summing up, our predictive capacity remained very low despite the identification of some of relevant factors associated with non-adherence.

Our study has some limitations. First, although our cohort includes all patients after a hip fracture from Valencia, a region covering a population of 5 million inhabitants, this population represents approximately 11% of the overall Spanish population. Thus, results should be extrapolated with caution, especially given that studies have shown a considerable variability in hip fracture rates and osteoporotic treatment between different regions, even within the same country^21,22^. Second, we calculate secondary non-adherence with dispensing but no prescription data, so we cannot discern whether treatment interruption is instructed by clinicians or is due to patient’s non-adherence. Additionally, we used pharmacy claims for measuring adherence, but there is no certainty that the patient actually consumes the medication filled from the pharmacy. Nevertheless, several studies have shown a high consistency between dispensation and patient consumption^23,24^. Those are common features in adherence studies. Third, we may be unable to document inpatient use of osteoporosis medication used inside hospital during the inpatient journey or in patients hospitalised during follow-up for reasons other than a new hip fracture, though this may only have a minor impact on our results. Additionally, we have potentially missed prescriptions of zoledronic acid, which is licensed as a hospital-use only product in Spain, but its uptake is very low in our setting and this may only affect our results marginally. Finally, regarding adherence predictors, some important factors related to adherence (e.g. factors related to healthy behaviours, caregivers or even health providers) may have not been considered in our study, a common issue when working with clinical data from administrative or electronic health records.

In conclusion, we found that the use of osteoporotic medication after a hip fracture is markedly low in the region of Valencia. When medication is prescribed, primary non-adherence is minor but secondary non-adherence is significant and increases sharply over time. Our study shows that despite the availability of effective, safe and affordable medications, and the unambiguous evidence-based directions for using those medications in secondary prevention of hip fracture patients, lack of medication adherence is concerning. As lower adherence is associated with a higher risk of recurrent fracture^25^, there is an urgent need to work to improve medication adherence to ameliorate the care we deliver to these patients.

## References

[1] Lips P, van Schoor NM (2005). Quality of life in patients with osteoporosis. Osteoporos Int..

[2] Sanfélix-Genovés J, Hurtado I, Sanfélix-Gimeno G, Reig-Molla B, Peiró S (2011). Impact of osteoporosis and vertebral fractures on quality-of-life. A population-based study in Valencia, Spain (The FRAVO Study). Health Qual Life Outcomes..

[3] Johnell O (2004). Mortality after osteoporotic fractures. Osteoporos Int..

[4] Hopkins RB (2016). The current economic burden of illness of osteoporosis in Canada. Osteoporos Int..

[5] Bliuc D (2009). Mortality Risk Associated With Low-Trauma Osteoporotic Fracture and Subsequent Fracture in Men and Women. JAMA..

[6] Azagra R (2015). Incidence of hip fracture in Spain (1997–2010). Med Clin (Barc)..

[7] Etxebarria-Foronda I (2015). Regional variability in changes in the incidence of hip fracture in the Spanish population (2000–2012). Osteoporos Int..

[8] Järvinen TL (2015). Overdiagnosis of bone fragility in the quest to prevent hip fracture. BMJ..

[9] Sanfélix-Genovés J (2014). Variability in the recommendations for the clinical management of osteoporosis. Med Clin (Barc)..

[10] Leslie WD (2012). A population-based analysis of the post-fracture care gap 1996–2008: the situation is not improving. Osteoporos Int..

[11] Rabenda V (2008). Low incidence of anti-osteoporosis treatment after hip fracture. J Bone Joint Surg Am..

[12] Díez-Pérez A (2011). Regional differences in treatment for osteoporosis. The Global Longitudinal Study of Osteoporosis in Women (GLOW). Bone..

[13] Kim SC (2015). Use of osteoporosis medications after hospitalization for hip fracture: a cross-national study. Am J Med..

[14] Osterberg L, Blaschke T (2005). Adherence to medication. N Engl J Med..

[15] Reynolds K (2013). Primary non-adherence to bisphosphonates in an integrated healthcare setting. Osteoporos Int..

[16] Beardon PH, McGilchrist MM, McKendrick AD, McDevitt DG, MacDonald TM (1993). Primary non-compliance with prescribed medication in primary care. BMJ..

[17] Andrade SE, Kahler KH, Frech F, Chan KA (2006). Methods for evaluation of medication adherence and persistence using automated databases. Pharmacoepidemiol Drug Saf..

[18] Sikka R, Xia F, Aubert RE (2005). Estimating medication persistency using administrative claims data. Am J Manag Care..

[19] Solomon DH (2014). Osteoporosis medication use after hip fracture in U.S. patients between 2002 and 2011. J Bone Miner Res..

[20] Sanfélix-Gimeno G (2013). Oportunidades de mejora en el abordaje de la osteoporosis. Tiempo de abordar lo importante. Med Clin (Barc)..

[21] Etxebarria-Foronda I (2015). Regional variability in changes in the incidence of hip fracture in the Spanish population (2000–2012). Osteoporos Int..

[22] Sanfélix-Genovés J, Peirò S, Sanfélix-Gimeno G, Hurtado I (2011). Prevalence of densitometric osteoporosis and osteopenia in Spain. Bone.

[23] Steiner JF, Prochazka AV (1997). The assessment of refill compliance using pharmacy records: methods, validity, and applications. J Clin Epidemiol..

[24] Grymonpre R, Cheang M, Fraser M, Metge C, Sitar DS (2006). Validity of a prescription claims database to estimate medication adherence in older persons. Med Care..

[25] Siris ES (2009). Impact of osteoporosis treatment adherence on fracture rates in North America and Europe. Am J Med..

